# Understanding the phenomenon of saltwater intrusion sourced from desalination plants at coastal aquifers

**DOI:** 10.1007/s11356-023-29866-y

**Published:** 2023-09-28

**Authors:** Farhat Abbas, Salem Al-Naemi, Aitazaz A. Farooque, Michael Phillips, Derek A. Rose

**Affiliations:** 1 College of Engineering and Technology, University of Doha for Science and Technology, P.O. Box 24449, Doha, Qatar; 2 Office of the President, University of Doha for Science and Technology, P.O. Box 24449, Doha, Qatar; 3 Canadian Centre for Climate Change and Adaptation, University of Prince Edward Island, Charlottetown, PE C1A 4P3 Canada; 4 Faculty of Sustainable Design Engineering, University of Prince Edward Island, Charlottetown, PE C1A 4P3 Canada; 5 Directorate of Applied Research, Innovation and Economic Development, University of Doha for Science and Technology, P.O. Box 24449, Doha, Qatar; 6 School of Agriculture, Food and Rural Development, University of Newcastle, Newcastle Upon Tyne, NE1 7RU UK

**Keywords:** Water treatment, Groundwater contamination, Miscible displacement, Surface-solute interactions, Breakthrough curves

## Abstract

Members of the Gulf Cooperation Council countries Bahrain, Kuwait, Oman, Qatar, Saudi Arabia, and the United Arab Emirates rely on desalination to produce water for domestic use. Desalination produces brine that may intrude into the aquifers to pollute the fresh groundwater because of the concentration gradient and groundwater pumping. Modeling the trends of saltwater intrusion needs theoretical understanding and thorough logical experimentation. The objective of this exercise was to understand the phenomenon of saltwater intrusion using an existing set of data analyzed with the convective–diffusion equation and the two-region mobile–immobile solution model. The objective was achieved by optimizing non-measurable solute transport parameters from an existing set of data generated from a series of logical miscible displacements of potassium bromide through sepiolite minerals and curve-fitting simulations. Assumptions included that solute displacements through sepiolite porous media and the related simulations represented the phenomenon of saltwater intrusion under non-equilibrium conditions of porous media mimicking the aquifers. Miscible displacements of potassium bromide were observed from a column of 2.0–2.8 mm aggregates of sepiolite over 4 ranges of concentration and at 11 displacement speeds under saturated vertical flow deionized water and vice versa. Breakthrough curves of both bromide and potassium ions were analyzed by a curve-fitting technique to optimize transport parameters assuming solute movement was governed (i) by the convective–diffusion equation and (ii) the two-region mobile–immobile solution model. Column Peclet numbers from the two analyses were identical for potassium ions but those for bromide ions were c. 60% greater from the two-region model than from the convective–diffusion equation. For the two-region model, dispersion coefficients were well defined and remained unchanged from the convective–diffusion equation for potassium ions but decreased for bromide ions. Retardation factors for bromide ions were approximately the same, but those for potassium ions, though > 1, were poorly defined. In order to design mitigation strategies for avoiding groundwater contamination, this study’s findings may help model groundwater pollution caused by the activities of desalination of seawater, which produces concentrated liquid that intrudes into the coastal aquifer through miscible displacement. However, robust saltwater intrusion models may be considered in future studies to confirm the results of the approach presented in this exercise. Field data on the groundwater contamination levels may be collected to compare with simulated trends drawn from the saltwater intrusion models and the curve-fitting technique used in this work. A comparison of the output from the two types of models may help determine the right option to understand the phenomena of saltwater intrusion into coastal aquifers of various characteristics.

## Introduction

Worldwide, desalination plants produce a huge amount of potable water. Desalination of seawater is actively practiced for human, industrial, and agricultural uses by countries in the Gulf Cooperation Council (GCC) countries, which include Bahrain, Kuwait, Oman, Qatar, Saudi Arabia, and the United Arab Emirates (Bilal et al. 2021). In addition to portable water, desalination also produces brine or saltwater that may intrude into the aquifers varyingly depending upon its concentration levels, the equilibrium/non-equilibrium conditions of structured aquifers, and excessive pumping of groundwater. Saltwater intrusion contaminates groundwater (Cuthbert et al. 2019), another source in GCC countries to fulfill drinking, domestic, and agricultural irrigation needs (Wu et al. 2020). Beyond the limit groundwater pumping, especially in the coastal regions, for irrigation or for any other use withdraws coastal freshwater and causes the inland flow of saltwater into fresh coastal aquifers (Goswami et al. 2020; Walther et al. 2020).

Avoiding groundwater contamination must be considered seriously as one-third of global water demands are fulfilled with groundwater (Famiglietti 2014). Qatar’s internal production of groundwater hardly reaches 56 million cubic meters (Mm^3^) per year with an annual consumption rate of 250 Mm^3^ mainly by agriculture (about 91%) (Alhaj et al. 2017). Qatar National Vision 2030 emphasizes a backup supply of irrigation-suitable water in abundance (QNV 2030). The limited availability of groundwater, low annual rainfall (< 80 mm) (AlMamoon et al. 2014), and huge per annum evaporation rate (2000 mm) (Darwish 2015) force Qatar’s authorities to fulfill the country’s water needs through desalination (QNV 2030). Desalination produces brine that may intrude into the aquifers to pollute the fresh groundwater because of the concentration gradient and groundwater pumping. Deep knowledge and efficient techniques to mitigate saltwater intrusion can results in contamination-free groundwater in coastal regions (Abd-Elaty et al. 2021, 2022a,b).

Desalination is a process of obtaining treated freshwater from brackish and/or saline seawater. It is a potential solution for water supply in many countries including Qatar, which have no or limited access to freshwater supplies (Elsaid et al. 2020). The global desalination capacity has increased from its 2005 estimates of ~ 35 to ~ 95 Mm^3^/day by the end of the second decade of the twenty-first century (Gleick 2006) with two-thirds of its consumption in municipalities and the remaining one-third in industrial operations (Jones et al. 2019). The Middle East/North Africa (MENA) including GCC countries dominantly produces and consumes about half of the world’s desalinated water followed by the Asia Pacific and North America having shares of about 18 and 12%, respectively (Jones et al. 2019). Qatar fulfills its 50% water needs from the desalination of its seawater (Baalousha and Ouda 2017).

As per common practices worldwide, the brine produced from desalination is injected into the saline groundwater (SGW) portion of a coastal aquifer posing two primary risks; (i) brine making its way back to the ocean under the phenomenon of submarine groundwater discharge and (ii) seawater (saltwater) intrusion or inland movement of the interface between fresh and saline groundwater (Stein et al. 2021). In the case of GCC countries, the seawater intrusion resulting from the injection of brine into the saline portion of the aquifer can pose a risk to already-scarce freshwater resources in areas requiring desalination by reverse osmosis (Stein et al. 2020). Saltwater intrusion is impacted by sea level rises (Werner and Simmons 2009; Shi et al. 2020), seasonal variations in the groundwater levels (Andersen et al. 2005; Michael et al. 2005; Yu and Michael 2019), occasional ocean surge events (Paldor and Michael 2021), and mainly by extensive pumping of fresh groundwater from coastal aquifers (Zhou et al. 2005; Stein et al. 2020).

The salinity level of the Arabian Gulf seawater exceeds 45 kg/m^3^ (Ahmed et al. 2018), in terms of total dissolved salts (TDS), in comparison to the seawater TDS range of 35 to 45 kg/m^3^ (WHO 2004). On average, seawater in the world’s oceans has a salinity of about 35 kg/m^3^ (Tiwari et al. 2014). However, seawater salinity in the west of Qatar and in some locations reaches 60 kg/m^3^ (Elsaid et al. 2020). As earlier said, desalination of such saline water produces concentrated brine that can intrude into coastal aquifers in the complex shapes of saltwater if not properly treated before disposing it back to the sea. Understanding the two-regions–based solute transport in a porous media under equilibrium and non-equilibrium states is vital for estimating groundwater/soil contamination resulting from saltwater intrusion (Khuzhayorov et al. 2020). The theoretical basis for novel mitigation strategies to avoid groundwater contamination from saltwater intrusion needs to be developed and/or evaluated. The novelty of the approach for conducting such investigations analytically strengthened by modeling output is obvious. Once estimated accurately, the procedure may be helpful in studying the economic dimension of water during determining the offset of the cost of water production from desalination plants while considering the indirect return from using this water and its environmental impacts (Abbas et al. 2023).

A large number of papers have been published in the past several years reporting on the simultaneous movement of water and solutes through porous media. The earliest workers discussed results in terms of the shape of the resulting breakthrough curves (BTCs) as one solution, initially resident in the porous medium, was displaced by a different solution. One of the initial quantitative analyses of the convective–diffusion equation (CDE) was cataloged by Peaceman and Rachford (1962). For the CDE analyses, applied to one-dimensional flow through a homogeneous medium at uniform water content, the solute transport is usually characterized by two independent parameters (retardation factor, *R*; dispersion coefficient, *K*). Such analyses assumed continuous concentration gradients everywhere within the medium—an equilibrium situation. These analyses were of differing degrees of subjectivity, one of the most objective being the use of the logarithmic–normal transform to convert sigmoid BTCs to lines whose slopes depended on the column Peclet number (van Genuchten and Wierenga 1986; Rose and Passioura 1971), which was applicable to both finite and semi-finite boundary conditions.

Subsequently, there was a second approach, that of transport through a medium with a bimodal pore-size distribution, in which micropores within structural units are separated by macropores through which solution flows — the two-region or mobile–immobile model of van Genuchten and Wierenga (1976): solute transport is described by 4 independent parameters, including *R* and *K*. This is a non-equilibrium situation because of differences in concentration between the micro- and macropore domains—a possible and assumed case of saltwater intrusion into the coastal freshwater (Parker and van Genuchten 1984). Passioura (1971), however, showed that solute movement through such aggregated materials could be described by the CDE over a wide range of displacement speeds and aggregate sizes by treating the micropores as a distributed source or sink, specifying the critical velocity at which the transition from equilibrium to non-equilibrium occurred (see also Rao et al. 1980).

Concurrently, the development of convenient software to analyze displacement experiments, such as CXTFIT (Abbas and Rose 2010; Toride et al. 1995), enabled the transport parameters to be simultaneously optimized from both the CDE and the two-region model, but only for semi-infinite boundary conditions. These parameters are then used to predict the fate of chemicals in the environment, often without much attention to the physicochemical mechanisms involved in their transport (see, for example, Vanderborght and Vereecken (2007) and references therein). There have, however, been relatively few critical comparisons of these two methods of analysis on extensive datasets. The contributions of the work, reported here, are the use of large sets of data from a series of miscible displacement experiments where the dense solutions (mimicking saltwater) replaced less dense liquids (mimicking fresh groundwater) and vice versa. Data generated from such analytical exercises are scientifically important and can be used to understand various real-world phenomena (such as the phenomenon of saltwater intrusion sourced from desalination plants at coastal aquifers), using the available curve-fitting techniques (CXTFIT in this case) that can estimate non-measurable variables mentioned in the “Materials and methods” section of this article. The intensive literature review did not reveal any study conducted and/or reported on the use of analytical experiments and the related simulation techniques to understand micro-level solute transport governing saltwater intrusion into coastal aquifers. This work is one of a kind to fulfill such a knowledge gap.

It is hypothesized that understanding CDE and its all parameters can help to model solute transport as a result of saltwater intrusion that pollutes the fresh groundwater aquifers. Therefore, the importance of this work is addressing the impacts of the scarce nature of water resources in the GCC countries due to the low average annual rainfall and high evaporation. Desalination plants that have been established to treat the seawater for substituting the water deficiency in GCC countries produce concentrated wastewater that may intrude the coastal aquifers. The transport of solutes carried by the intruding water can be studied analytically and theoretically to model certain parameters that are impossible to measure through experimentation. The novelty of this work includes determining such optimized parameters from the use of the existing datasets generated from the equilibrium and non-equilibrium analyses of miscible displacement experiments. Therefore, the objective of this exercise was to understand the phenomenon of saltwater intrusion using an existing set of data analyzed with the convective–diffusion equation and the two-region mobile–immobile solution model. Assumptions included that solute displacements through sepiolite porous media and the related simulations represented the phenomenon of saltwater intrusion under non-equilibrium conditions of porous media mimicking the geology of GCC countries aquifers (Al-Sarawi 1995; Sherif and Hamza 2001; Melaine et al. 2008; Koussis et al. 2010; Abbas et al. 2023). The study used an existing extensive dataset (Abbas 2001) that was generated from conducting a series of analytical experiments in the UK.

## Materials and methods

### Theory

The theory of solute transport through a porous material is well established and has been developed from first principles by, among others, Van Genuchten and Wierenga (1976), Toride et al. (1995), and Nkedi-Kizza et al. (1983). The equations that are relevant to this study are used for equilibrium and non-equilibrium analyses. For equilibrium analysis of the one-dimensional steady flow at a pore-water velocity, *v* = *q*/*θ*_*t*_ where *q* is the Darcy flux and *θ*_*t*_ is the total porosity, the CDE for fluid flow under longitudinal hydrodynamic dispersion through a column of a uniform, saturated, and reactive porous material of length *L* is

$$R\frac{\partial C}{\partial t}=K\frac{{\partial }^{2}C}{\partial {x}^{2}}-v\frac{\partial C}{\partial x}$$

in which.

*C*: dimensionless concentration of solute = (*c* − *c*_*i*_)/(*c*_*i*_ − *c*_*o*_).
*c*: concentration of solute
*c*_*i*_: displacing concentration of solute.
*c*_*o*_: resident concentration of solute.
*x*: longitudinal coordinate.
*t*: time.
*K*: coefficient of hydrodynamic dispersion.
*R*: retardation factor = 1 + (*k*_*d*_* ρ*/*θ*_*t*_), assumed to be constant over the concentration interval (*c*_*i*_ − *c*_*o*_).

The form of the breakthrough curve is determined by the column Peclet number (Rose and Passioura 1971), *P* = *vL*/*K*. The CDE applies to solute transport through aggregates (Passioura 1971) provided that the pore-water velocity does not exceed a maximum, *v*_*max*_. However, the pore space of aggregates is partitioned into micropores within structural units, in which liquid is essentially stagnant, separated by macropores through which liquid flows. Solute transport is within and through such aggregates by radial diffusion within the microporosity (the immobile solution) and by hydrodynamic dispersion in the macroporosity (the mobile solution). A non-equilibrium analysis is used for such two-region models to define immobile and mobile solutions. Transport models are based on the first-order exchange of solute between the mobile and immobile water regions. van Genuchten and Wierenga (1976) extended this concept of mobile-immobile water to include the Freundlich-type equilibrium adsorption process where the solute transport is assumed to occur as a first-order exchange between the two regions of solution as

$${\theta }_{\text{m}}{R}_{\text{m}}\frac{\partial {c}_{\text{m}}}{\partial t}={\theta }_{\text{m}}K\frac{{\partial }^{2}c}{\partial {x}^{2}}-{v}_{\text{m}}{\theta }_{\text{m}}\frac{\partial {c}_{\text{m}}}{\partial x}-{\theta }_{\text{im}}{R}_{\text{im}}\frac{\partial {c}_{\text{im}}}{\partial t}$$

$${\theta }_{\text{im}}{R}_{\text{im}}\frac{\partial {c}_{\text{im}}}{\partial t}=\alpha \left({c}_{\text{m}}-{c}_{\text{im}}\right)$$

The values of the parameters of two-region models including *a* (mass transfer rate coefficient between the mobile and immobile regions), *c*_*m*_ (solute concentration in the mobile solution), *c*_*im*_ ( solute concentration in the immobile solution), *θ*_*m*_ (volume of mobile solution as a proportion of the total column volume), *θ*_*im*_ (volume of immobile solution as a proportion of total column volume), and *R*_*m*_ and *R*_*im*_ (dimensionless retardation factors, which account for equilibrium-type adsorption in the mobile and immobile regions, respectively) may be recovered from dimensionless forms of the two-region models. The dimensionless parameters include *β* (a partitioning coefficient), *ω* (a dimensionless mass-transfer coefficient), *f* (the fraction of adsorption sites within the mobile-water region), and ϕ (the ratio of mobile to the total volume of fluid).

An estimate for *f* is needed in order to calculate $\varphi$, because there is no independent measurement for *f*. Estimates of *f* have included *f* = ϕ (Nkedi-Kizza et al. 1983), ϕ/2 (Seyfried and Rao 1987), zero (Nkedi-Kizza et al. 1982), and 1 (Selim and Liwang 1995). Unfortunately, as pointed out by Nkedi-Kizza et al. (1983), the values of *f* and ϕ cannot be obtained from a single displacement experiment because both are included in *β*. Nkedi-Kizza et al. (1984) discussed these four approaches in detail and concluded that one could simply assume *f* to be zero if any reaction occurred only in the stagnant region of the soil, i.e., within the aggregates. This assumption appears realistic for materials in which the interior surface area of the aggregates is much larger than the exterior surface area, e.g., the aggregated sepiolite used in experiments of this study.

Numerous models have been proposed and tested in the literature (see Huyakorn et al. 1987; Mantoglou 2003; Datta et al. 2009; Meyer et al. 2019 and the references therein) to study the phenomena of saltwater intrusion into coastal aquifers of various geological formations. The models used in this study have been chosen based on their simplicity to use, comprehension of two-region situations, the capability of representing concentrated liquids’ transport through mobile-immobile water under equilibrium and non-equilibrium conditions, and feasibility of curve fitting techniques (CXTFIT) to fit models on the experimental BTCs to estimate the required non-measurable variables. Analyses of these data with non-specific saltwater intrusion models may be considered a limitation of this study.

### Experimental

**Fig. 1:**
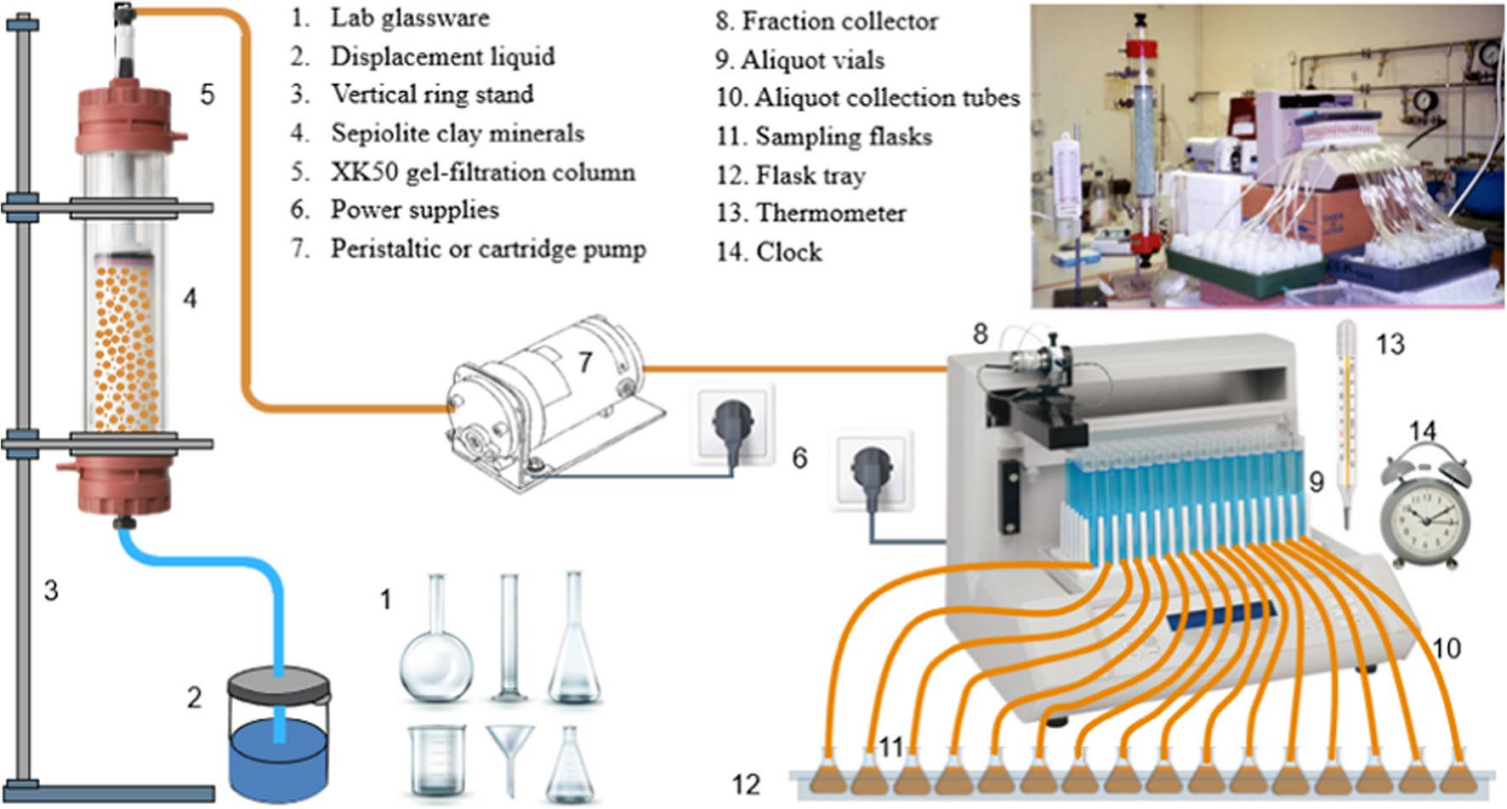
A schematic and a reference photo of the experimental setup (Abbas 2001). Since an original photo of a sepiolite-packed gel-filtration column could not be found, a sample photo showing a glass ballotini-filled gel-filtration column in the same series of analytical experiments is presented. Note that in this series of analytical trials, various media were used for miscible displacement experiments including glass ballotini, sepiolite clay minerals, and porous pumice stones

The porous material was sepiolite, a magnesium silicate clay mineral from Vallescas in Spain, supplied as porous aggregates (Berk Mineral Products, Worksop S81 7QQ, UK). This sepiolite has a cation exchange capacity of 0.26 mol_*c*_ kg^−1^ = 25.2 kC kg^−1^, and a total (internal plus external) surface area of 330 m^2^ g^−1^ giving a density of surface charge of 0.79 μmol_*c*_ m^−2^ = 0.076 C m^−2^ (Robertson 1957). The aggregates retain their geometry and physical structure as the composition and concentration of the saturating solution alter. A sieved fraction (2.0–2.8 mm) was packed uniformly into an XK50 gel-filtration column (Amersham Pharmacia Biotech, Little Chalfont, Bucks HP7 9AA, UK) with a diameter of 50 mm to a length of 29.6 cm (Fig. 1). The pistons (flow adaptors) of this column are designed to provide uniform flow at the entrance and exit of the bed of porous material and a minimal dead volume (less than 1% of the total column volume). The column was saturated under a vacuum with deionized water and remained saturated with liquid throughout the experiments.

The total porosity, *θ*_*t*_, was 0.798 m^3^ m^−3^, found by successively weighing the column empty, packed with sepiolite dried over silica gel, and saturated with deionized water under vacuum, allowing for the water in the dead volume. The microporosity, *θ*_*a*_, was 0.392 m^3^ m^−3^, found from the water content of initially saturated aggregates at a suction of 75 cm (Khuzhayorov et al. 2020). The macroporosity was then 0.406 m^3^ m^−3^, and the internal porosity of the aggregates was 0.66 m^3^ m^−3^. The internal porosity of an aggregate is the ratio of the total volume of pores in the aggregate to the total volume of the aggregate. It is denoted by *ε*_*I*_, expressed in cm^3^ cm^−3^, and measured for sepiolite. For this, the aggregates were washed, cleaned, and oven dried and then saturated under vacuum with de-aired de-ionized water. They were then removed from the water, wiped, cleaned of surplus surface water, weighed (*W*_*sag*_), and oven dried for 24 h then weighed again (*W*_*dag*_), where *W*_*sag*_ and *W*_*dag*_ stand for the weight of saturated and oven-dry aggregates, respectively. Assuming the aggregates were initially saturated, the volume of pores within the aggregates was assumed to be equal to the mass of water content divided by the density of water. The aggregate internal porosity was calculated as follows:

The volume of water content (*V*_*w*_) which is considered to be equal to the volume of pores within the aggregates was measured as

$${V}_{w} = ({W}_{sag}- {W}_{dag}) / {\rho }_{w}$$

where *V* is volume and *W* is weight. The subscripts _*w*_, _*sag*_, and _*dag*_ refer to water, saturated aggregates, and dry aggregates respectively, and ρ_w_ (0.998 g cm^−3^) is the density of water at the experimental temperature (20 °C).

The volume of the dry solid matrix (*V*_*d*_) is

$${V}_{d}= {W}_{dag}/{\rho }_{p}$$

where ${\rho }_{p}$ is the particle density of aggregates. The volume of the aggregates (*V*_*ag*_) is given by

$${V}_{ag} = {V}_{w} + {V}_{d}$$

and aggregate internal porosity (*ε*_*I*_) by

$${\varepsilon }_{I} = {V}_{w}/{V}_{ag}$$

The total pore volume (*V*_*p*_) of the sepiolite-packed columns was 464 cm^3^, which for a porous medium is the ratio of the total volume of pores in the medium to the total volume of the medium (Nielsen & Biggar 1962). It was calculated from the internal porosity of the column (*ε*_*T*_) and volume of the medium (*V*_*m*_) as

$${V}_{p}= {\varepsilon }_{T} {V}_{m}$$

Solutions of analytical grade potassium bromide (KBr) at 4 concentrations (10, 3, 1, and 0.6 mM) were used. Concentrations of bromide ion (Br^–^) and potassium ion (K^+^) were measured with ion-specific electrodes which were frequently calibrated using 4 or 5 standard solutions over the particular range of concentration used in the displacements. Displacements were thus at average concentrations, $\overline{c }$, of 5, 1.5, 0.5, and 0.3 mM because no K^+^ eluted from sepiolite in deionized water could be detected below 0.3 mM. These four ranges of concentration have been termed as A, B, C, and D, respectively.

The experiments involved deionized water displacing a KBr solution initially resident in the vertical column of saturated sepiolite, and vice versa. Eleven pore-water velocities ranging from 0.89 to 198 cm h^–1^, 3 of which exceeded the limit (*c*. 50 cm h^–1^) above which the CDE fails to apply to the sepiolite (calculated following Passioura 1971), were used. The displacements were controlled at the lower velocities by a cartridge pump (Model 7553–85, Cole Parmer Instruments, Chicago, IL 60648, USA) and at the higher velocities by a standard peristaltic pump (type MHRE, Watson-Marlow Bredel Pumps, Falmouth TR11 4RU, UK).

The effluent was collected with a fraction collector (type FC203B, Gilson, 3000 West Beltane Highway, Middleton, WI 53562, USA) modified to accept volumes of up to 80 cm^3^ per aliquot. The usual aliquot volume was 35 cm^3^, and displacements were continued until 3 pore volumes of liquid had been collected. All experiments and chemical analyses were done at a temperature of 20 ± 1 °C. These miscible displacement experiments were conducted during the study reported by Abbas (2001).

The displacements were all repeated in reverse. Thus, for each concentration and displacement speed, first water displaced solution (leaching, desorption of solute), and then solution displaced water (uptake, resalinization, sorption of solute). The sequence of the experiments was as follows: (i) displacements at the highest solution concentration were completed before starting those at the next highest concentration, and so on; (ii) for a given concentration, displacements proceeded from slowest to fastest, with each immediately repeated in reverse; (iii) alternate experiments involved deionized water displacing solution resident in the porous material and vice versa, irrespective of the speed of displacement. The data of these alternate experiments (category iii) when deionized water displaced concentrated KBr solution resident in sepiolite column was used for this report in order to represent a saltwater intrusion into an aquifer of fresh water.

### Data analysis

Non-dimensional breakthrough curves (BTCs) for the concentrations of the individual ions (Br–, K +) for each displacement were produced. The BTCs were analyzed with the appropriate solutions to CDE for the equilibrium and non-equilibrium analyses in CXTFIT 2.1 (Baalousha and Ouda 2017) for the time course of the flux concentration of each ion in the effluent at the exit of the column following an initial step-change in concentration at the entrance to the column. For the CDE, only the value of the pore-water velocity *v* = *q*/*θ*_*t*_ was specified as that delivered by the calibrated pump to control flow. Thus, a free optimization, for each ion in each displacement, of the values of *R* and *K* in the equilibrium, 2-parameter, and CDE formulation of solute transport was allowed.

For the physical, non-equilibrium model, the input files of CXTFIT require initial values of the parameters (*R*, *K*, *β*, and *ω*) to be specified. These values are crucial to the outcome of the optimization and so need to be fixed accurately. As equilibrium was achieved in each displacement (Rose et al. 2009), initial values were set as the optimized values of *R* and *K* under equilibrium conditions. The initial values for *β* and *ω* were fixed at 0.9 and 0.02, respectively, because the general solution for non-equilibrium transport reduces to that for equilibrium transport when *β* = 1 and *ω* = 0 (Toride et al. 1993).

## Results

_*E*_ and _*N*_ refer to results from equilibrium and non-equilibrium analyses, respectively. The equilibrium results are all based on the optimization of 16 BTCs; the non-equilibrium results on various numbers (*n*) as some BTCs proved unquantifiable by CXTFIT, and some optimized values were discarded as unreliable. In the tables, results are presented as mean values ± standard deviations: coefficients of variation, *σ* = (standard deviation ÷ mean) × 100%.

### Reversibility

All displacements were reversible irrespective of the type of analysis. The BTCs and dispersion coefficients by regressing log *P* or log *K* for leaching against the corresponding values for resalinization were tested: in every case, the slope did not differ significantly from unity or the intercept from zero. Similarly, there were no significant differences between values of *R*, *β*, $\varphi$, and α from leaching and resalinization over a given concentration interval when assessed by paired *t*-tests. Accordingly, the results from the leaching and resalinization BTCs were combined to increase *n*.

### Breakthrough curves

**Fig. 2:**
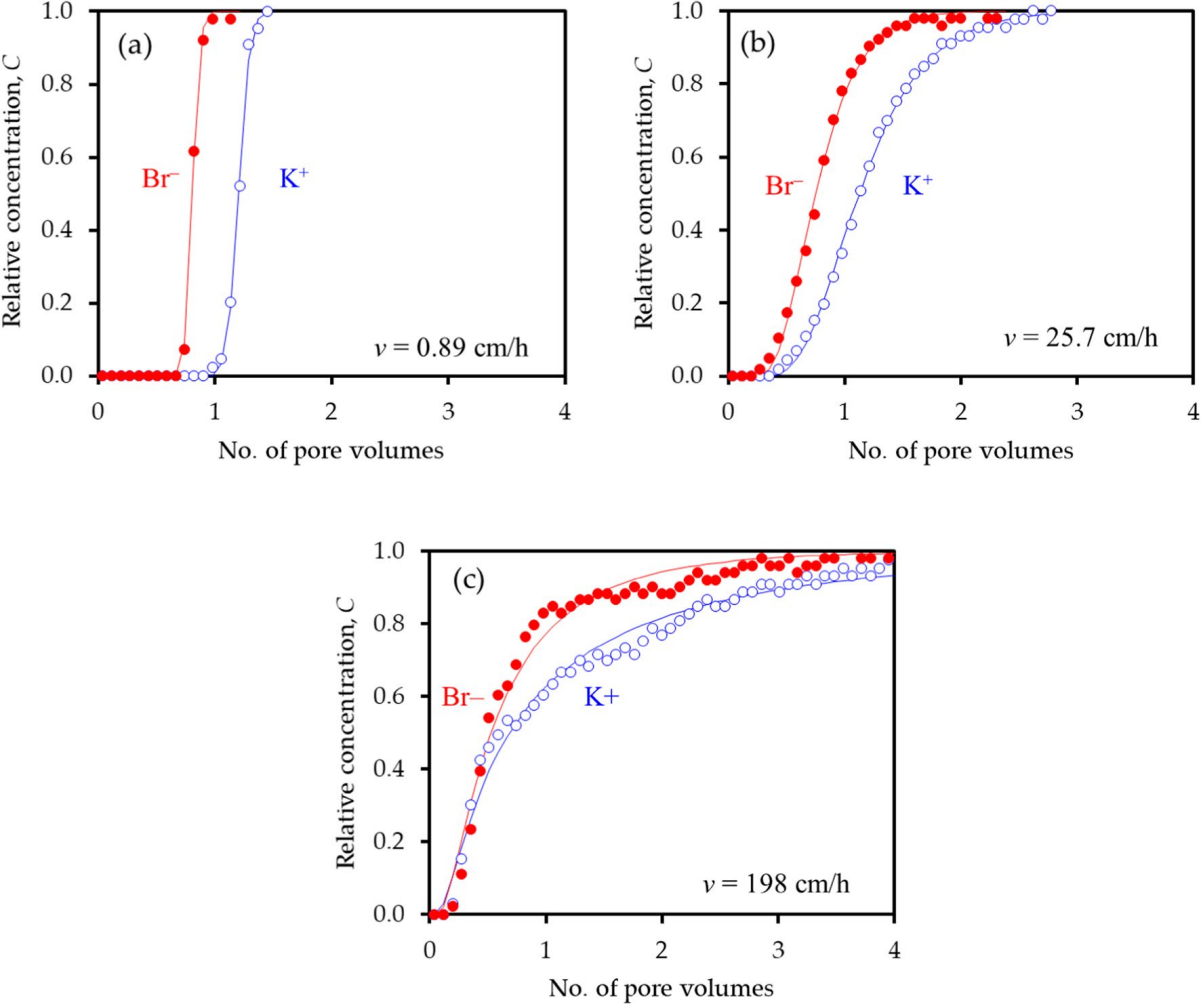
Breakthrough curves for Br^–^ (filled red circle) and K^+^ (open blue circle) at 3 representative pore-water velocities including **a** *v* = 0.89 cm/h, **b** *v* = 25.7 cm/h, and **c** *v* = 198 cm/h during the displacement of water by 0.6 Mm KBr solution

Figure 2 shows measured and fitted BTCs for the individual ions during the displacement of water by 0.6 mM KBr solution at 3 representative pore-water velocities: the fastest displacement in Fig. 2c exceeds *v*_*max*_. The BTCs were well fitted by CXTFIT, with the goodness-of-fit value of *R*^2^ averaging 99.75 ± 0.12% and rarely falling below 99.5%. The form of the BTCs is determined by the column Peclet number, *P*.

**Fig. 3:**
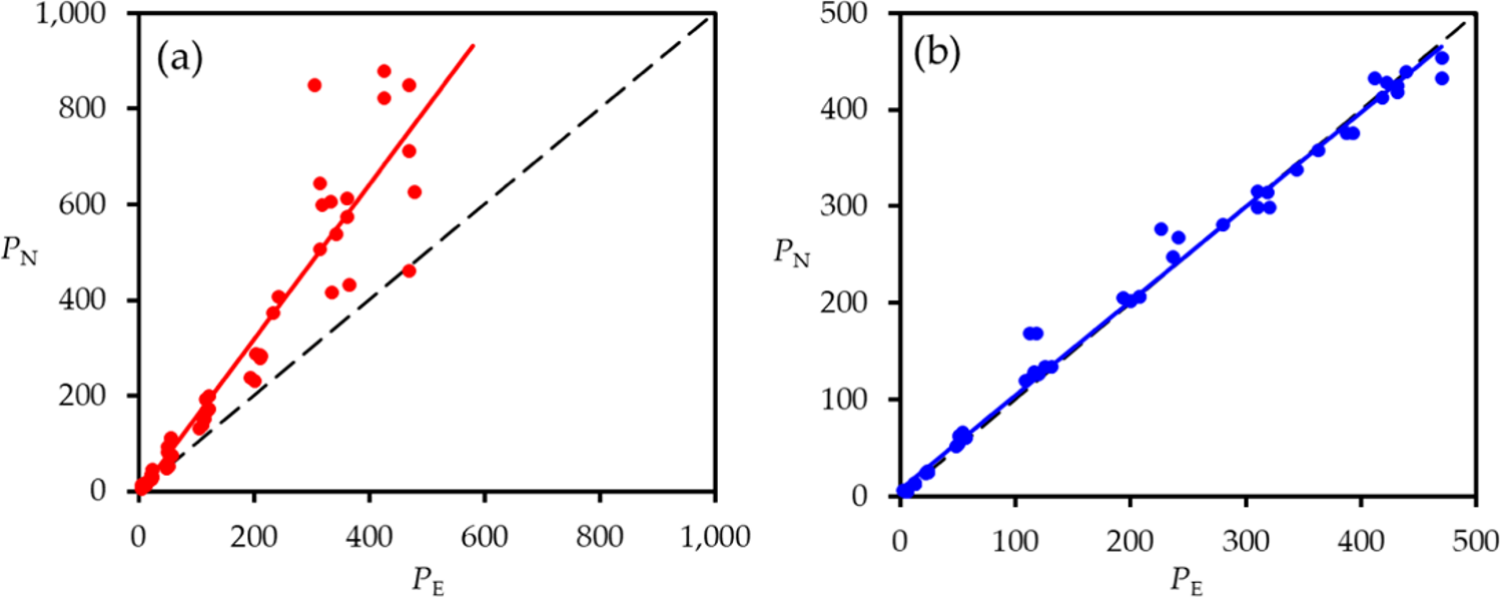
Comparison of column Peclet numbers from non-equilibrium, *P*_*N*_, and equilibrium, *P*_*E*_, analyses for **a** Br^–^ and **b** K^+^. The regression lines are solid; the 1:1 lines are dashed

Figure 3 compares estimates from the non-equilibrium analyses, *P*_*N*_, with those from the corresponding equilibrium analyses, *P*_*E*_.

For Br^–^ (Fig. 3a), *P*_*N*_ increases from *c*. 6–7 at the fastest displacement to *c*. 850 at the slowest, consistently exceeding *P*_*E*_ which ranges between *c*. 4–5 and *c*. 500. Although there is some scatter in the data, particularly relating to the steeper BTCs, the regression is highly significant:

$${P}_{N} = 1.624 {P}_{E}- 7.97, r = 0.951, n = 63, p < 0.001$$

In contrast, for K^+^ (Fig. 3b), the estimates from the two methods of analysis are comparable:

$${P}_{N} = 0.977 {P}_{E}+ 6.46, r = 0.996, n = 64, p < 0.001$$

The consequences of these outcomes are manifested in the estimates of dispersion coefficients — see later.

At the highest velocities, when *v* > *v*_*max*_, the equilibrium analyses failed (Rose et al. 2009), so comparisons between the two methods of analysis cannot be made. In addition, the values of *P*_*E*_ were then consistently less than 5, so optimizations by CXTFIT of the CDE were inappropriate for a finite column because they are strictly only valid for semi-infinite boundary conditions. Nkedi-Kizza et al. (1983) observed *P*_*N*_ > *P*_*E*_ for both ^36^Cl and ^3^H_2_O, though the scatter in their data was much more extreme than in this investigation. In addition, the majority of their BTCs had *P*_*E*_ < 5 whereas the majority of their non-equilibrium analyses had *P*_*N*_ > 5.

### Retardation factor, R

Table 1 compares values of the retardation factor estimated by the two methods of analysis for the four ranges of concentration in the displacements. *R*_*E*_ was independent of velocity and closely defined with average coefficients of variation, $\tilde{\sigma }$, of 0.7% for Br^–^ and 0.4% for K^+^. Values of *R*_*E*_ were < 1 for Br^–^ and > 1 for K^+^ and became more extreme as concentration decreased. For Br^–^, *R*_*N*_ was also less than 1 and decreased as concentration decreased, though *R*_*N*_ values exceed *R*_*E*_ in each concentration range and are less well defined ($\tilde{\sigma }$ = 1.6%). For K^+^, *R*_*N*_ exceeds unity but is poorly defined ($\tilde{\sigma }$ = 8.2%) and independent of concentration. For both ions, *R*_*N*_ was independent of velocity.

**Table 1:** Mean values and standard deviations of retardation factor optimized from equilibrium, *R*_*E*_, and non-equilibrium, *R*_*N*_, analyses. *n*_*E*_ = 14-16

Ion	Range	*R*_*E*_	*R*_*N*_	*n*_*E*_
Bromide	A	0.9314 ± 0.0046	0.9441 ± 0.0169	14
	B	0.8989 ± 0.0067	0.9145 ± 0.0094	16
	C	0.8510 ± 0.0052	0.8657 ± 0.0138	16
	D	0.8083 ± 0.0063	0.8270 ± 0.0159	15
Potassium	A	1.0631 ± 0.0019	1.2313 ± 0.1373	15
	B	1.1120 ± 0.0024	1.2116 ± 0.0819	16
	C	1.1515 ± 0.0053	1.3390 ± 0.1224	16
	D	1.1963 ± 0.0100	1.2682 ± 0.0720	16

### Dispersion coefficient, D

**Fig. 4:**
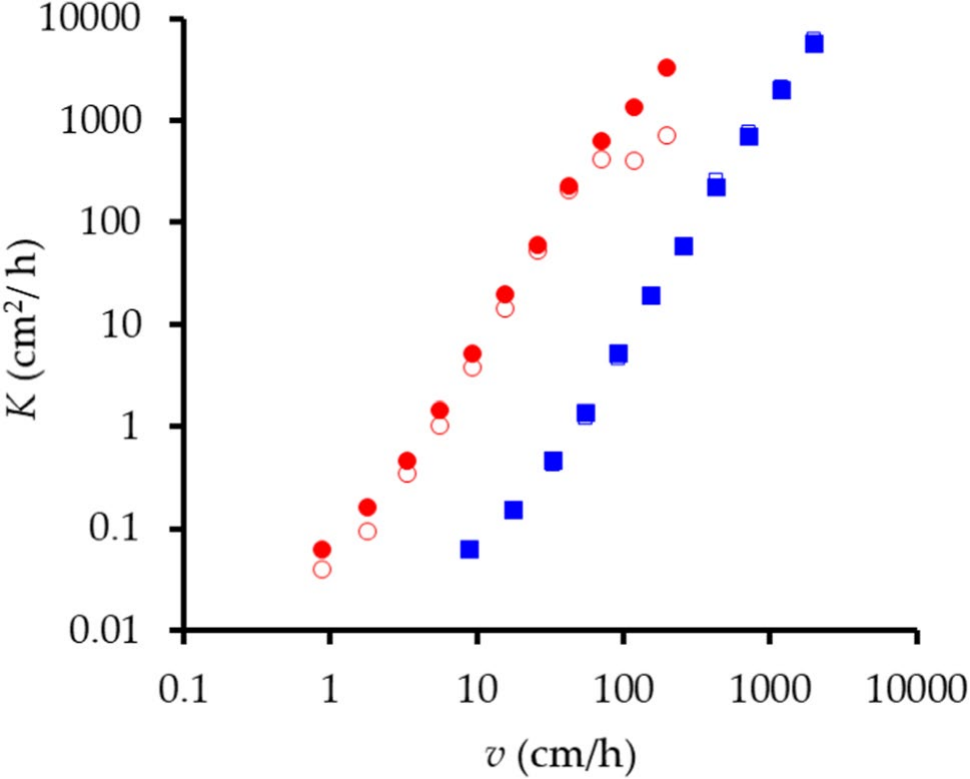
Coefficients of hydrodynamic dispersion, *K* cm^2^/h, as functions of pore-water velocity, *v* cm/h. Open symbols, non-equilibrium analysis; closed symbols, equilibrium analysis. Br^–^ open and filled red circle; K^+^ open and filled blue squares. The data points for K^+^ have been shifted by one decade to the right to avoid overlap

Figure 4 shows values of *K* as functions of pore-water velocity, *v*, including the 3 values when *v* > *v*_*max*_. The data points for K^+^ have been shifted one decade to the right to avoid overlap because the functions *K*_*E*_ (*v*) for Br^–^ and K^+^ are congruent within experimental error. Each point is the mean of 8 values (leaching and resalinization in each concentration range) as a GLM analysis confirmed that there were no significant effects of concentration on *K*. Mean values are well defined ($\tilde{\sigma }$ of 6–8%) except for *K*_*N*_ (Br^–^) with $\tilde{\sigma }$ = 20%.

For Br^–^, *K*_*N*_ < *K*_*E*_ at all velocities, but differences were small for K^+^, a consequence of the differing behaviors of *P*_*E*_ and *P*_*N*_ for the two ions (Fig. 4). All results were well fitted by equations of the form *K* = *a* + *bv* + *cv*^2^ applicable to aggregated systems when *v* < *v*_*max*_ (Passioura et al. 1971) but diverged from this at larger speeds, especially for *K*_*N*_ (Br^–^). It is nevertheless difficult to discern significant trends from Fig. 4 or comparisons of the regression coefficients in the fitted parabola.

**Fig. 5:**
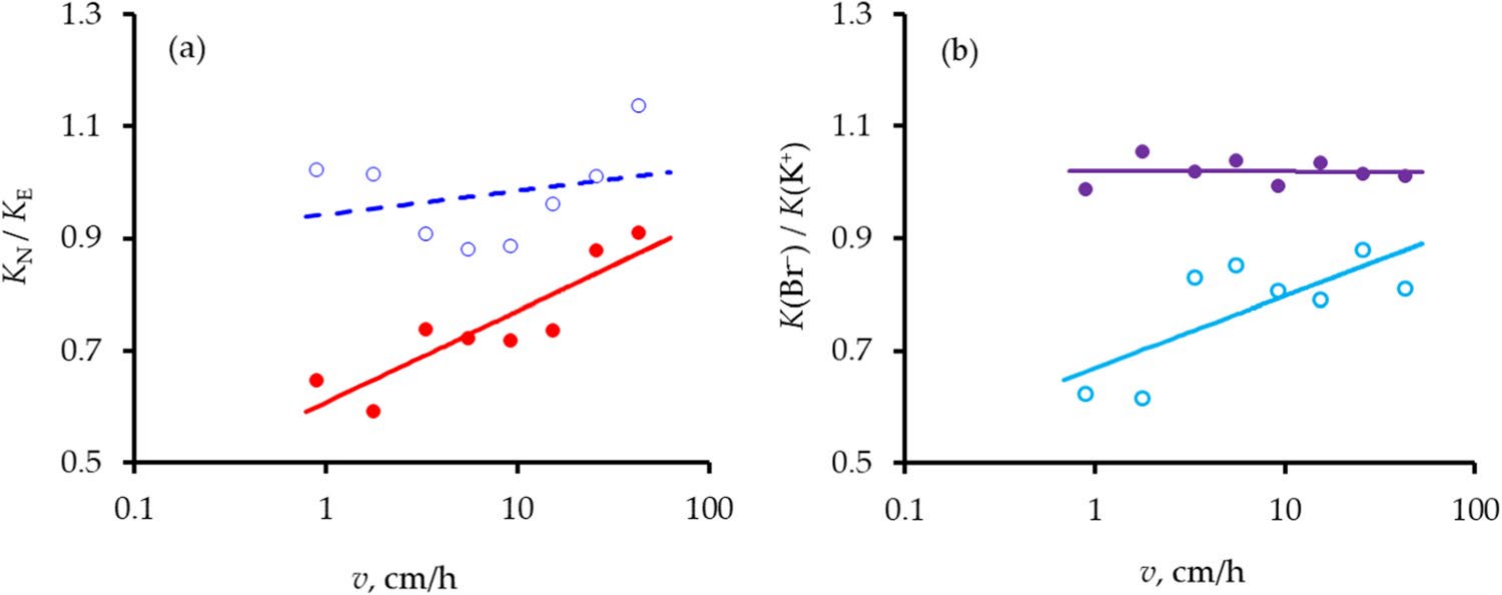
Ratio of values of dispersion coefficient **a** from non-equilibrium, *K*_*N*_, and equilibrium, *K*_*E*_, analyses as functions of pore-water velocity, *v*, for Br^–^ (filled red circle) and K^+^ (open blue circle) and **b** of Br^–^, *K*(Br^–^), to that of K^+^, *K*(K^+^), for equilibrium (filled purple circle) and non-equilibrium (open sky blue circle) analyses

Figure 5 a, however, compares ratios of the values of *K* from the two methods of analysis as functions of *v*. There is a significant increase in *K*_*N*_/*K*_*E*_ as *v* increases for Br^–^ (*r* = 0.89, *p* < 0.01) but not for K^+^ (*r* = 0.28). Figure 5 b compares the ratio of the dispersion coefficients of the two ions as functions of *v*. There is a significant increase in this ratio as *v* increases for the non-equilibrium analyses (*r* = 0.74, *p* < 0.05) but not for the equilibrium analyses (*r* =  − 0.01): for the latter, the ratio was constant at 1.019 ± 0.023. These differences between ions and methods of analysis are, however, small compared to the effect of speed of displacement on *K*.

### Parameters derived from non-equilibrium analysis

Table 2 presents values of the optimized parameters and derived variables from the non-equilibrium analyses as discussed below.

**Table 2:** Mean values and standard deviations of derived parameters from the non-equilibrium analyses

Range	A	B	C	D
Ion	Bromide (Br^–^)			
*n*	14	16	16	15
*R*_*N*_	0.9441 ± 0.0169	0.9145 ± 0.0094	0.8657 ± 0.0138	0.8270 ± 0.0159
*β*	0.9529 ± 0.0292	0.9618 ± 0.0224	0.9360 ± 0.0337	0.9606 ± 0.0215
*ɸ*	0.9069 ± 0.0175	0.8794 ± 0.0138	0.8103 ± 0.0326	0.7941 ± 0.0099
*ω*	0.1940 ± 0.0620	0.1680 ± 0.1270	0.1880 ± 0.0730	0.0830 ± 0.0740
*θ*_*m*_	0.7237 ± 0.0139	0.7018 ± 0.0100	0.6466 ± 0.0260	0.6337 ± 0.0079
*θ*_*im*_	0.0743	0.0962	0.1514	0.1643
*/R*_*E*_	0.974	0.978	0.952	0.982
Ion	Potassium (K^+^)			
*n*	15	16	16	16
*R*_*N*_	1.2313 ± 0.1373	1.2116 ± 0.0819	1.3390 ± 0.1224	1.2682 ± 0.0720
*β*	0.8739 ± 0.0896	0.9246 ± 0.0567	0.8724 ± 0.0899	0.9425 ± 0.0499
*ɸ*	1.0648 ± 0.0157	1.1159 ± 0.0190	1.1589 ± 0.0520	1.1921 ± 0.0259
*ω*	0.0110 ± 0.0070	0.028 ± 0.0230	0.0090 ± 0.0040	0.0630 ± 0.0740
*θ*_*m*_	0.8497 ± 0.0125	0.8905 ± 0.0152	0.9248 ± 0.0415	0.9513 ± 0.0206
*θ*_*im*_	− 0.0517	− 0.0925	− 0.1268	− 0.1533
*/R*_*E*_	1.002	1.004	1.006	0.996

The standard deviations are equal to those for *θ*_*m*_

#### Relative partitioning factor, β

Parameter *β* accounts for partitioning of transport between phases 1 and 2 (Toride et al. 1993) which constitute the equilibrium (i.e., mobile) and non-equilibrium (immobile) parts of the transport system, respectively. When *β* is small, leaching is less effective at removing solute from soil. As *β* increases, conditions in phase 2 increasingly resemble those in phase 1; more of the transport occurs in phase 1 and leaching becomes more efficient: the limiting case of equilibrium transport occurs when *β* = 1.

Values of *β* were *c*. 0.95 for Br^–^ and *c*. 0.90 for K^+^ and independent of concentration. Although *β* was more closely defined for Br^–^ than for K^+^, there was a small but significant decrease in *β* as *v* increased for Br^–^ but not for K^+^. The data of Nkedi-Kizza et al. (1984) also show *β* decreasing not only as the speed of displacement increased but also as concentration decreased, though the scatter in their results is large. Bajracharya and Barry (1997), however, show that *β* is independent of *v*.

#### Proportion of pore space in mobile phase, ϕ

In Table 2, the values of ϕ, like those of *R*_*E*_, are less than unity for Br^–^, more than unity for K^+^, and become more extreme as concentration decreases. Although individual values of *β* and *R*_*N*_ vary widely for K^+^, those optimized from a particular BTC self-compensate so that ϕis estimated with some precision. At first sight, a value of ϕ > 1 implies that the macroporosity exceeds the total porosity, which is absurd. Averaging over the four ranges of concentration, however, the ratio ϕ*/R*_*E*_ = 1.002 ± 0.004 for K^+^, so that ϕ appears to equate to the retardation factor from the equilibrium analysis for the cation. For Br^–^,ϕ < *R*_*E*_ with ϕ/*R*_*E*_ = 0.972 ± 0.013 suggesting that $\varphi$ and *R*_*E*_ are significantly distinct.

#### ***Mobile and immobile volumetric water contents, θ***_***m***_*** and θ***_***im***_

These are simply calculated as *θ*_*m*_ = $\varphi$ *θ*_*t*_ and *θ*_*im*_ = *θ*_*t*_ − *θ*_*m*_. For Br^–^, following from the behavior of ϕ, *θ*_*m*_ decreases and *θ*_*im*_ increases as concentration decreases. This is physically realistic, but *θ*_*m*_ greatly exceeds the volumetric liquid content of the macroporosity, 0.406 m^3^ m^–3^, and *θ*_*im*_ is less than that of the microporosity, *θ*_*a*_ = 0.392 m^3^ m^–3^, of the column of sepiolite aggregates. For K^+^, *θ*_*m*_ increases and *θ*_*im*_ decreases as concentration decreases, but *θ*_*m*_ exceeds the total porosity, *θ*_*t*_, and *θ*_*im*_ is negative, which is physically unrealistic, but which follows from the rigorous application of the non-equilibrium optimization.

#### ***Volume of exclusion, θ***_***ex***_

**Fig. 6:**
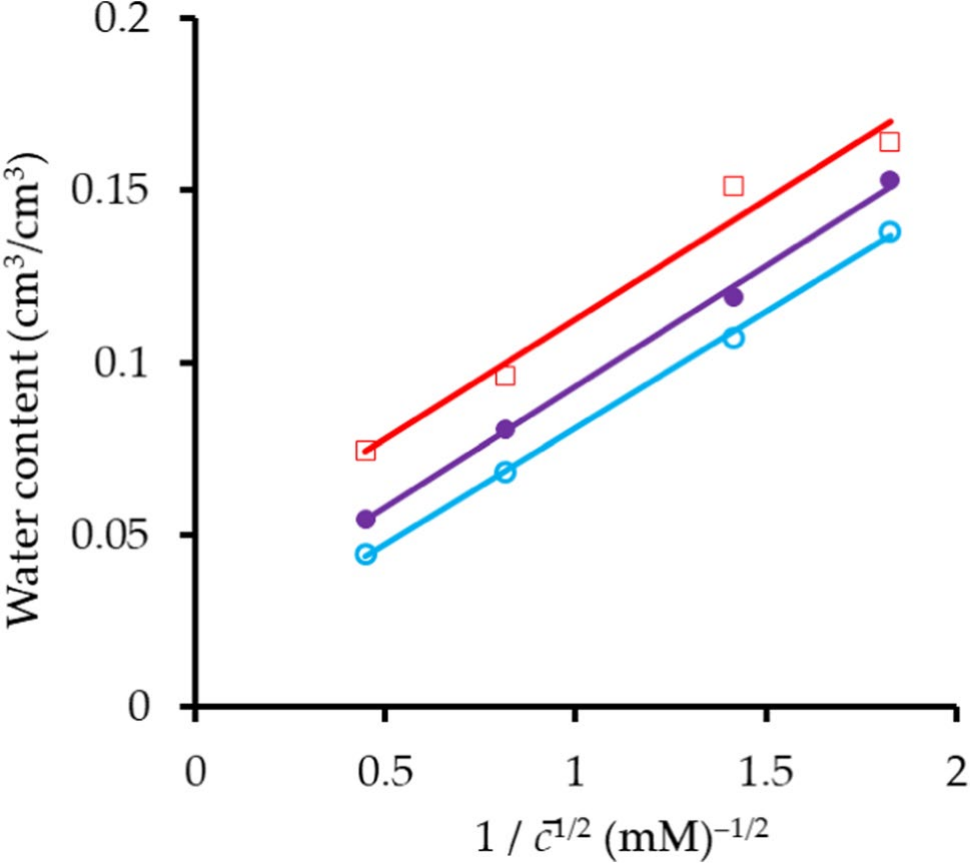
Effect of average concentration on volumes of immobile and excluded solution for Br^–^. *θ*_*im*_ open red square, *θ*_*ex*_ non-equilibrium open sky blue circle, *θ*_*ex*_ equilibrium filled purple circle

This is calculated as *θ*_*ex*_ = (1 − *R*)*θ*_*t*_ for the anion and is the volumetric liquid content adjacent to the clay surfaces from which anions are excluded by the electrical double layer. Values are not tabulated but displayed in Fig. 6 as functions of 1/$\overline{c }$ ^1/2^, where $\overline{c }$ is the average concentration over a displacement. The results for *θ*_*im*_ are also graphed. Each line is statistically significant with *p* < 0.05 for *θ*_*im*_, *p* < 0.01 for (*θ*_*ex*_)_*E*_, and *p* < 0.001 for (*θ*_*ex*_)_*N*_. The slopes are similar, but the extrapolated intercepts on the abscissa differ 0.043 m^3^ m^–3^ for *θ*_*im*_, 0.023 m^3^ m^–3^ for (*θ*_*ex*_)_*E*_, and 0.014 m^3^ m^–3^ for (*θ*_*ex*_)_*N*_. These results thus accord with the simple equation of Bolt and de Haan (1979) for the thickness of the electrical double layer adjacent to a plane surface of a negatively charged clay in a dilute solution of a single salt composed of monovalent anions and cations (thickness proportional to *c*^–1/2^). The simple equation does not hold at higher concentrations so that extrapolations to infinite concentration (*c*^–1/2^ = 0) have little physical significance.

#### Mass-transfer coefficients, ω and α

For Br^–^, CXTFIT gave definite estimates of *ω* in 59 of 64 optimizations when *v* < *v*_*max*_, and average values (Table 2) ranged between 0.1 and 0.2 with large coefficients of variation and little effect of concentration. There was a trend of decreasing *ω* as *v* increased in each concentration range, statistically significant in the three lowest concentration ranges. As a consequence of the wide range of displacement velocities, however, the mass-transfer coefficient α increased as *v* increased, typically from less than 0.01 h^–1^ to 0.05–0.10 h^–1^ over the range of *v* < *v*_*max*_.

For K^+^, *ω* was poorly defined with estimates from only 37 optimizations. Average values increased from *c*. 0.01 in range A to 0.06 in range D. There was no discernable effect of velocity on *ω* and α, and the mean value of α was 0.006 ± 0.008 h^–1^. When *v* > *v*_*max*_, *ω* was not fitted for K^+^. For Br^–^, however, values ranging between 0.2 and 0.8 were optimized leading to values of *α* between 0.3 and 1.4 h^–1^, the limited data not permitting effects of velocity and concentration to be explored. Nkedi-Kizza et al. (1984) found that *ω* was the most inconsistent parameter in their optimizations, influenced by the aggregate size, flux, and concentration. Rao et al. (1980) had earlier pointed out that *ω* was not constant for a given ionic species but a function of aggregate size, flux, and mobile-water content.

#### ***Distribution coefficients, k***_***d***_

**Fig. 7:**
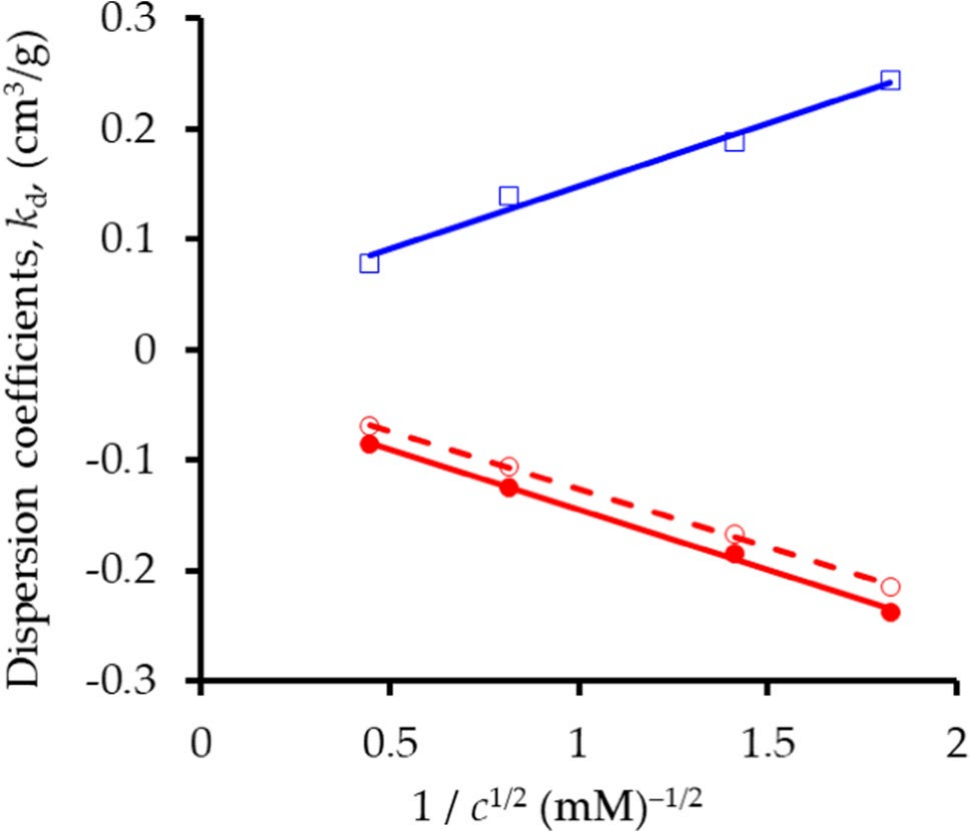
Distribution coefficients, *k*_*d*_, as functions of solution concentration, *c*. *k*_*E*_ (K^+^) open blue square, *k*_*E*_(Br^–^) open red circle, *k*_*N*_ (Br^–^) filled red circle

Distribution coefficients, *k*_*d*_, follow directly from optimized values of *R* and the physical characteristics of the system (bulk density, *ρ* = 0.642 g cm^–3^; *θ*_*t*_ = 0.798 cm^3^ cm^–3^). They are negative (exclusion) for the anion, positive (adsorption) for the cation, and the moduli of the values from the equilibrium analyses are comparable. Figure 7 demonstrates that they are linear functions of 1/*c*^1/2^, with *p* < 0.01, over the range of dilute concentrations in these displacements, whose slopes are comparable. The values for Br^–^ from the non-equilibrium analyses are larger (i.e., *less* negative) than those from the equilibrium analyses, but no reliable values for K^+^ can be derived because of the unreliable estimates of *R*_*N*_ (Table 1). Rose et al. (2009) recovered complete Freundlich isotherms for Br^–^ and K^+^ in sepiolite from the equilibrium estimates of *k*_*d*_, but this is not repeated here.

### *Representations of*$\varphi$

Table 3 contains estimates of $\phi$ from the four different assumptions of *f*, the proportion of reaction sites in the mobile-water region, for each concentration range. For each ion, $\phi$ was most closely defined when *f* = 0: for other formulations, the values for Br^–^ were much better defined than those for K^+^. All estimates for Br^–^ were less than unity, increasing in each concentration range in the order *f* = 0 < ϕ/2 < ϕ < 1: most estimates for K^+^ were less than unity (but not when *f* = 0), decreasing in each concentration range in the order *f* = 0 > ϕ/2 > ϕ>1. As noted earlier (Table 2 and text), with *f* = 0, ϕ decreased continuously for Br^–^ and increased continuously for K^+^ as concentration decreased. For the other formulations, there was little effect of concentration when *f* = 1 and *f* = ϕ, but overall decreases (Br^–^) and increases (K^+^) when *f* = ϕ/2. From this behavior, it can be concluded that *f* = 0 is the most appropriate assumption for our aggregated sepiolite.

**Table 3:** Comparative values (mean ± standard deviation) of ϕ for different assumptions of the fraction of exchange sites assumed to be at equilibrium, *f*

Ion	Range	0	ϕ/2	ϕ	1
Bromide	A	0.907 ± 0.017	0.929 ± 0.021	0.953 ± 0.029	0.955 ± 0.029
	B	0.879 ± 0.014	0.919 ± 0.017	0.962 ± 0.022	0.967 ± 0.020
	C	0.810 ± 0.033	0.869 ± 0.033	0.936 ± 0.034	0.945 ± 0.029
	D	0.794 ± 0.010	0.869 ± 0.013	0.961 ± 0.021	0.967 ± 0.019
Potassium	A	1.065 ± 0.016	0.958 ± 0.059	0.874 ± 0.090	0.834 ± 0.141
	B	1.116 ± 0.019	1.010 ± 0.036	0.925 ± 0.058	0.904 ± 0.079
	C	1.159 ± 0.052	0.994 ± 0.071	0.872 ± 0.090	0.820 ± 0.136
	D	1.192 ± 0.026	1.052 ± 0.035	0.943 ± 0.050	0.924 ± 0.071

Researchers (van Genuchten and Wierenga 1986; Toride et al. 1995) argued that *f* = 0 was the best formulation for an aggregated system, but nevertheless used *f* = ϕin their studies of ^3^H_2_O, ^36^Cl, and ^45^Ca in an aggregated Oxisol.

## Discussion

Despite reports that groundwater contamination/salinization is caused by agricultural, industrial, domestic, and urban development activities (Benaafi and Al-Shaibani 2021; Frollini et al. 2022), Kloppmann et al. (2013) found that groundwater was salinized by saltwater intrusion in the French aquifers they investigated. Examining a volcano-sedimentary aquifer in Djibouti’s coastal region of Africa, Ahmed et al. (2017) also concluded that saltwater intrusion and other geogenic processes were responsible for groundwater deterioration. Field observations have been reported in the literature to study the pattern of saltwater intrusion in different aquifers. Theoretical and analytical approaches have not been extensively used for this purpose. This study is an effort to optimize non-measurable solute transport parameters (Tables 1, 2, and 3) using an existing set of data generated from a series of logical miscible displacements through sepiolite minerals and curve-fitting simulations to understand and estimate patterns of saltwater intrusion.

The equilibrium analyses invariably gave excellent definitions to both *R*_*E*_ and *K*_*E*_, though the uncertainty was less in the former than in the latter (Rose et al. 2009). The non-equilibrium analyses, however, despite the excellent fits to the BTCs, often delivered extremely wide confidence ranges to and/or unrealistic values of some of the fitted parameters. These excellent fits often resulted from mutual compensation among the 4 optimized parameters leading to the poor identification of some or all of them and a consequent inability to identify the process correctly and unambiguously. In general, *K*_*N*_ was better defined than *R*_*N*_, particularly for the cation (Table 1; Fig. 4), for which *K*_*N*_ = *K*_*E*_. This reflects the need for using specialized techniques and/or models representing saltwater intrusion into coastal aquifers of specific geology. For example, Abdalla (2016) studied a portion of Jazan aquifer (southwest of Saudi Arabia) to understand the impact of seawater intrusion and to assess the dominant hydrogeochemical processes controlling groundwater pollution. Field data comprising 70 groundwater samples were analyzed for the chemistry of major cations and anions using conservative tracer methods and assumptions of Appelo and Postma (2004), Slama (2010), and Zghibi et al. (2013). It was recommended to establish a seawater intrusion monitoring well network followed by periodic monitoring of groundwater concentration using electrical conductivity particularly near the coastal area to track salinization. Lathashri and Mahesha (2015) used SEAWAT-2000 a coupled version of MODFLOW and MT3DMS to conceptually simulate groundwater flow and transport for a coastal stretch in Karnataka state (India) and recommend to be scientifically sound for further saltwater and groundwater management applications.

In order to reduce the uncertainty in estimating these non-equilibrium parameters, three more restricted options, following the example of Bajracharya and Barry (1997), were tried:

1. Fixing *R*_*N*_ at the overall mean *R*_*E*_ (Table 1) for a set of measurements from the equilibrium analyses, e.g., all 16 displacements at *v* < *v*_*max*_ over a given concentration range, and allowing free optimization of *K*_*N*_, *β* and *ω*
2. Fixing *K*_*N*_ at the mean value of *K*_*E*_ from corresponding leaching and uptake displacements at the same velocity, and free optimization of *R*_*N*_, *β* and *ω*
3. Fixing both *R*_*N*_ and *K*_*N*_ as in (1) and (2) above, and free optimization of *β* and *ω*

Table 4 contains some outcomes from these modifications for displacements in the lowest concentration range, and from the original free fits. With each option, *β* increased towards unity, ϕ remained stable, and *ω* decreased towards zero. With option (1), dispersion coefficients, *K*_*N*_, increased by an average of 10% for Br^–^ but were unchanged for K^+^. With option (2), values of *R*_*N*_ were unchanged for both ions. Overall, the modifications achieved little; such techniques to guide optimization, in fact, bias the outcome and are not recommended, although apparently used successfully by Nkedi-Kizza et al. (1984) and Bajracharya and Barry (1997).

**Table 4:** Mean values and standard deviations of parameters optimized from modified non-equilibrium analyses for lowest concentration range *D*

Ion	Option	*β*	ϕ	*ω*
Bromide	Free	0.960 ± 0.025	0.796 ± 0.009	0.083 ± 0.074
	(i)	0.988 ± 0.014	0.799 ± 0.011	0.039 ± 0.033
	(ii)	0.980 ± 0.023	0.810 ± 0.007	0.020 ± 0.014
	(iii)	0.991 ± 0.009	0.801 ± 0.008	0.025 ± 0.019
Potassium	Free	0.951 ± 0.042	1.184 ± 0.018	0.063 ± 0.074
	(i)	0.985 ± 0.013	1.179 ± 0.016	0.064 ± 0.081
	(ii)	0.951 ± 0.048	1.195 ± 0.013	0.010 ± 0.013
	(iii)	0.994 ± 0.006	1.189 ± 0.008	0.034 ± 0.030

The probable reason that the non-equilibrium model was relatively unsuccessful is the lack of usable data points on our BTCs of the dispersing front. A typical steep BTC might have 30 data points, only 7 of which were effectively used by CXTFIT, as data points with relative concentrations of zero and unity are ineffective in the optimization. In contrast, the non-equilibrium analysis has been used successfully by Nkedi-Kizza et al. (1983; 1984) and many others who used a pulse of solute rather than a front in their displacements. Typical BTCs from their work typically contain 25–30 data pairs, all of which are available to the model for identifying the process. It would be preferable in the future to use finite pulses of tracer rather than fronts for the most precise identification of transport parameters. This would apply to both equilibrium and non-equilibrium analyses, but especially the latter to better accommodate the free fitting of 4 independent parameters.

Passioura and Rose (1971) confirmed the applicability of the CDE to aggregated systems and the criterion for its breakdown in 43 displacements through 2 sizes of each of 3 inert and reactive materials with ^36^Cl (chlorine) as a tracer. Rao et al. (1980) explored the applicability of both models in 11 experiments with inert solid and aggregated materials and with ^36^Cl and ^3^H_2_O (water) as the tracers. Nkedi-Kizza et al. (1983) used both models to analyze the behavior of a pulse of ^36^Cl in tritiated CaCl_2_ (calcium chloride) solution from 17 displacements through aggregates of an Oxisol maintained at pH 7 (3 sizes × 2 velocities × 3 concentrations). Bajracharya and Barry (1997) examined the physical significance of non-equilibrium solute-transport parameters in 27 experiments with 3 materials (mixtures of porous polyethylene cylinders and either sand or silt), supplemented by numerical simulations. Rose et al. (2009) investigated the role of the electrical double layer on the surface–solute interactions affecting solute transport. They performed 230 displacements through 4 different materials with several ranges of concentration of solutions of 4 simple salts. One component of this investigation was the displacement of KBr solutions of 4 different concentrations through a column of 2.0–2.8 mm aggregates of sepiolite at 11 pore-water velocities.

Nkedi-Kizza et al. (1983) found the equilibrium analysis applied only to their small (0.5–1.0 mm) aggregates but failed with the larger (1–2 and 2–4.7 mm) sizes even at low pore-water velocities between 0.4 and 6 cm h^–1^. This was because they used short columns (5 cm long) which caused the CDE to fail even at such small displacement speeds because, theoretically, *v*_*max*_ is proportional to column length (Passioura 1971). The CDE, however, gave stable values of *R*_*E*_ and *K*_*E*_ for our 2.0–2.8-mm aggregates up to *v* = 42.8 cm h^–1^ and failed only when *v* exceeded its predicted limit of validity.

There is, however, no a priori reason why a non-equilibrium analysis should not apply within the equilibrium transport regime because it comprises an alternative intellectual construct, that of a pore architecture of two discrete regions each with distinct characteristics. This model, for the anion Br^–^, delivers plausible parameter values of both volumes of immobile solution and of anion exclusion consistent with electrical double-layer theory; although dispersion coefficients are modified, the effect is trivial compared to that of displacement speed. For the cation K^+^, dispersion coefficients are unchanged, but retardation factors and adsorption are poorly defined, and the partitioning of the pore space is physically unrealistic. Therefore, *R*_*E*_ and *K*_*E*_ for equilibrium analysis that may dominate the scenarios of saltwater intrusion into fresh aquifers can be easily determined by conducting analytical experiments on the specific porous media and the available model of CDE.

After understanding the scale and fate of pollutants moving from saltwater of sea to the groundwater, the former can be prevented to mix with the latter through conventional methods, physical barriers, and hydraulic barriers (Hussain et al. 2019; Abd-Elaty et al. 2021, 2022a,b). The negative hydraulic barrier that uses barrier wells to pump the brackish and/or saltwater has been advocated in literature (Ozaki et al. 2021) with an option of its reuse in desalination plants (Stein et al. 2019). Benaafi et al. (2022) assessed groundwater salinization in the shallow and deep coastal aquifers of the Al-Qatif region (an eastern region of Saudi Arabia) caused by saltwater intrusion and concluded that reduced withdrawal of groundwater in the central part of the study area raised its level by about three meters protecting it from saltwater intrusion. They advocated such management practices in the entire region and continuous monitoring of groundwater levels for informed decision-making.

## Conclusions

Analytical experimentation to understand the phenomena of miscible displacement that governs saltwater intrusion into the fresh coastal aquifers was conducted, and the results were discussed for behaviors of anions and cations under the equilibrium and non-equilibrium conditions individually and for with their interactions. The results are presented for debate as a contribution to the continuing problem of determining realistic parameter values for use in simulating solution transport in porous media. A major snag with formal optimization techniques, in general, is that the outcome can vary with the choice of the initial values of the parameters to be optimized, and the more parameters that are involved, the greater the opportunity for mutual adjustments among them. This is a particular drawback with non-equilibrium transport and a way forward might be to use a modeling technique such as ORCHESTRA (Meussen 2003), a framework for implementing chemical equilibrium and transport models, in which all parameter values are either known or can be independently measured and not need to be optimized. This technique has been used successfully by several workers to quantify the transport of solutes in reactive soils, e.g., van Beinum (2007) and references therein. It has also been successful in modeling the non-equilibrium leaching of ions from inert porous spheroids in a series of displacements in which the macropore and micropore solutions were separately labeled and traced (Abbas 2001; Rose et al. 2008).

Considering the mentioned assumptions, i.e., the current choice of models for their simplicity to use, comprehension of two-region situations, the capability of representing concentrated liquids’ transport through mobile-immobile water under equilibrium and non-equilibrium conditions, the feasibility of curve fitting techniques (CXTFIT) to fit models on the experimental BTCs to estimate the required non-measurable variables, and the limitations for this study, the robust saltwater intrusion models may be considered in the future to analyze these sets of data. Comparative assessments of the output from the two types of models thereafter may help recommend the most feasible method to define the phenomena of saltwater intrusion into coastal aquifers resembling the geology of GCC countries. One way of mitigation of groundwater water contamination of GCC was suggested by Al-Rashed and Sherif (2000), i.e., recycling of water and artificial recharge of groundwater by surface water and treated wastewater at a larger scale. Knowledge-based approaches can mitigate saltwater intrusion in coastal regions (Abd-Elaty et al. 2021, 2022a,b). Such solutions and knowledge, if applicable, will need careful investigation of the state of groundwater pollution to determine the scale of mitigation efforts. The novelty of the study reported here is the protocol for conducting such investigations. It may be helpful in studying the economic dimension of water during determining the offset of the cost of water production from desalination plants while considering the indirect return from using this water and its environmental impacts (Abbas et al. 2023).

## Data Availability

The data used for analysis are available upon a request from the corresponding author.

## Index of Acronyms, Notations, and Symbols

Br^–^: Bromide ion
BTC: Breakthrough curve
CaCl_2_: Calcium chloride
^36^Cl: Chlorine tracers
CDE: Convective–diffusion equation
CXTFIT: Curve fitting technique
GCC: Gulf Cooperation Council
^3^H_2_O: Water tracers
K^+^: Potassium ion
KBr: Potassium bromide

## Notations (units)

*c*: Concentration of solute (mol L^−^^3^)
*C*: Dimensionless concentration of solute (unitless)
*c*_*i*_: Displacing concentration of solute (mol L^−^^3^)
*c*_*im*_: Intra-aggregate “immobile water region” solute concentration (mol L^−^^3^)
*c*_*m*_: Inter-aggregate “mobile water region” solute concentration (mol L^−^^3^)
*c*_*o*_: Resident concentration of solute (mol L^−^^3^)
ƒ: Fraction of adsorption sites within the mobile-water region (unitless)
*K*: Hydrodynamic dispersion coefficient (L^2^ T^−^^1^)
*k*_*d*_: Empirical distribution coefficient (L^3^ M^−^^1^)
*L*: Length of column (L)
*P*: Peclet number (unitless)
*P*_*E*_: Peclet number from equilibrium analysis (unitless)
*P*_*N*_: Peclet number from non-equilibrium analysis (unitless)
*q*: Darcy flux (L T^−^^1^)
*R*: Retardation factor (unitless)
*R*_*E*_: Retardation factor from equilibrium analysis (unitless)
*R*_*im*_: Retardation factor in immobile region (unitless)
*R*_*m*_: Retardation factor in mobile region (unitless)
*R*_*N*_: Retardation factor from non-equilibrium analysis (unitless)
*t*: Time (T)
*v*: Average pore-water velocity (L T^−^^1^)
*v*_*max*_: Maximum pore-water velocity (L T^−^^1^)
*V*_*ag*_: Volume of dry aggregates (L^3^)
*V*_*d*_: Volume of dry solid mix (L^3^)
*V*_*m*_: Volume of medium (L^3^)
*V*_*p*_: Pore volume (L^3^)
*V*_*w*_: Volume of water content (L^3^)
*W*_*dag*_: Weight of oven dried (24 h) aggregates (M)
*W*_*sag*_: Weight of saturated aggregates (M)
*x*: Distance (M)

## Symbol

*α*: Mass-transfer coefficient (T^−^^1^)
*β*: Relative partitioning factor (unitless)
*ε*_*I*_: Internal porosity of particle (L^3^ L^−^^3^)
*ε*_*T*_: Total porosity (L^3^ L^−^^3^)
*ϕ*: Ratio of mobile to the total volume of fluid (unitless)
*ϕ*_*im*_: Fraction of immobile water content (unitless)
*ϕ*_*m*_: Fraction of mobile water content (unitless)
*ω*: Mass transfer coefficient (unitless)
*ρ*: Density (M L^−^^3^)
*ρ*_*b*_: Bulk density (M L^−^^3^)
*ρ*_*w*_: Density of water (M L^−^^3^)
*θ*_*a*_: Microporosity (L^3^ L^−^^3^)
*θ*_*im*_: Volume of immobile water content (L^3^ L^−^^3^)
*θ*_*m*_: Volume of mobile water content (L^3^ L^−^^3^)
*θ*_*t*_: Total volumetric water content in a medium/porosity (L^3^ L^−^^3^)
*σ*: Standard deviation (unit of the variable)
$\tilde{\sigma }$: Average coefficients of variation (unit of the variable)

## References

[CR1] Abbas F (2001) The effect of surface-solute interactions on the transport of solutes through porous media, PhD thesis, University of Newcastle upon Tyne. UK

[CR2] Abbas F, Rose DA (2010). Viscous fingering and gravity segregation: experimental results. Earth Interact.

[CR3] Abbas F, Al-Naemi S, Farooque AA, Phillips M (2023). A review on the water dimensions, security, and governance for two distinct regions. Water.

[CR4] Abdalla F (2016). Ionic ratios as tracers to assess seawater intrusion and to identify salinity sources in Jazan coastal aquifer, Saudi Arabia. Arab J Geosci.

[CR5] Abd-Elaty I, Straface S, Kuriqi A (2021). Sustainable saltwater intrusion management in coastal aquifers under climatic changes for humid and hyper-arid regions. Ecol Eng.

[CR6] Abd-Elaty I, Kuriqi A, Bhat SA, Zelenakova M (2022). Sustainable management of two-directional lateral and upcoming saltwater intrusion in coastline aquifers to alleviate water scarcity. Hydrol Process.

[CR7] Abd-Elaty I, Kushwaha NL, Grismer ME, Elbeltagi A, Kuriqi A (2022). Cost-effective management measures for coastal aquifers affected by saltwater intrusion and climate change. Sci Total Environ.

[CR8] Ahmed AH, Rayaleh WE, Zghibi A, Ouddane B (2017). Assessment of chemical quality of groundwater in coastal volcano-sedimentary aquifer of Djibouti, Horn of Africa. J African Earth Sci.

[CR9] Ahmed M, Kumar R, Al-Wazzan Y, Garudachari B, Thomas JP (2018). Assessment of performance of inorganic draw solutions tested in forward osmosis process for desalinating Arabian Gulf seawater. Arab J Sci Eng.

[CR10] Alhaj M, Mohammed S, Darwish M, Hassan A, Al-Ghamdi SG (2017). A review of Qatar’s water resources, consumption and virtual water trade. Desalin Water Treat.

[CR11] AlMamoon A, Joergensen NE, Rahman A, Qasem H (2014). Derivation of new design rainfall in Qatar using L-moment based index frequency approach. Int J Sustain Built Environ.

[CR12] Al-Rashed MF, Sherif MM (2000). Water resources in the GCC countries: an overview. Water Resour Manage.

[CR13] Al-Sarawi MA (1995). Surface geomorphology of Kuwait. GeoJournal.

[CR14] Andersen MS, Nyvang V, Jakobsen R, Postma D (2005). Geochemical processes and solute transport at the seawater/freshwater interface of a sandy aquifer. Geochim Cosmochim Acta.

[CR15] Appelo CAJ, Postma D (2004). Geochemistry, groundwater and pollution.

[CR16] Baalousha HM, Ouda OK (2017). Domestic water demand challenges in Qatar. Arab J Geosci.

[CR17] Bajracharya K, Barry DA (1997). Non-equilibrium solute transport parameters and their physical significance: numerical and experimental results. J Contam Hydrol.

[CR18] Benaafi M, Al-Shaibani A (2021). Hydrochemical and isotopic investigation of the groundwater from Wajid Aquifer in Wadi Al-Dawasir. Southern Saudi Arabia Water.

[CR19] Benaafi M, Tawabini B, Abba SI, Humphrey JD, Al-Areeq AM, Alhulaibi SA, ... Aljundi IH (2022) Integrated hydrogeological, hydrochemical, and isotopic assessment of seawater intrusion into coastal aquifers in Al-Qatif Area, Eastern Saudi Arabia. Molecules 27(20):6841 10.3390/molecules27206841 PMC9609279 36296433

[CR20] Bilal H, Govindan R, Al-Ansari T (2021). Investigation of groundwater depletion in the state of Qatar and its implication to energy water and food nexus. Water.

[CR21] Bolt GH, De Haan FMA (1979) Anion exclusion in soil. p. 233–256. In Bolt GH (ed) Soil Chemistry: B. Physico-Chemical Methods: Elsevier Scientific Publishing Company, Amsterdam

[CR22] Cuthbert MO, Taylor RG, Favreau G (2019). Observed controls on resilience of groundwater to climate variability in sub-Saharan Africa. Nature.

[CR23] Darwish MA (2015). Desalination engineering.

[CR24] Datta B, Vennalakanti H, Dhar A (2009). Modeling and control of saltwater intrusion in a coastal aquifer of Andhra Pradesh, India. J Hydro-Environ Res.

[CR25] Elsaid K, Kamil M, Sayed ET, Abdelkareem MA, Wilberforce T, Olabi A (2020). Environmental impact of desalination technologies: a review. Sci Total Environ.

[CR26] Famiglietti JS (2014). The global groundwater crisis. Nat Clim Chang.

[CR27] Frollini E, Parrone D, Ghergo S, Masciale R, Passarella G, Pennisi M, Salvadori M, Preziosi E (2022). An integrated approach for investigating the salinity evolution in a Mediterranean coastal karst aquifer. Water.

[CR28] Gleick PH (2006). The World’s Water 2006–2007: The Biennial Report on Freshwater Resources.

[CR29] Goswami G, Basack S, Mastorakis N, Saikia A, Nilo B, Ahmed N (2020). Coastal groundwater flow and management: a state-of-the-art review. Int J Mech.

[CR30] Hussain MS, Abd-Elhamid HF, Javadi AA, Sherif MM (2019). Management of seawater intrusion in coastal aquifers: a review. Water.

[CR31] Huyakorn PS, Andersen PF, Mercer JW, White HO (1987). Saltwater intrusion in aquifers: development and testing of a three-dimensional finite element model. Water Resour Res.

[CR32] Jones E, Qadir M, van Vliet MTH, Smakhtin V, Kang S (2019). The state of desalination and brine production: a global outlook. Sci Total Environ.

[CR33] Khuzhayorov B, Mustofoqulov J, Ibragimov G, Md Ali F, Fayziev B (2020). Solute transport in the element of fractured porous medium with an inhomogeneous porous block. Symmetry.

[CR34] Kloppmann W, Bourhane A, Schomburgk S (2013). Groundwater salinization in France. Procedia Earth Planet Sci.

[CR35] Koussis AD, Georgopoulou E, Kotronarou A, Lalas DP, Restrepo P, Destouni G, ... Gomez-Gotor A (2010) Cost-efficient management of coastal aquifers via recharge with treated wastewater and desalination of brackish groundwater: general framework. Hydrological Sciences Journal 55(7):1217–1233.

[CR36] Lathashri UA, Mahesha A (2015). Simulation of saltwater intrusion in a coastal aquifer in Karnataka, India. Aquatic Procedia.

[CR37] Mantoglou A (2003) Pumping management of coastal aquifers using analytical models of saltwater intrusion. Water Resour Res 39(12)

[CR38] Melaine ME, Alkhaddar RM, Phipps D (2008) Sustainable wastewater treatment packaged plants: energy use minimisation for small sites and for remote operation. In Liverpool Conference on the Built Environ Nat Environ 108

[CR39] Meussen JCL (2003). ORCHESTRA: an object-oriented framework for implementing chemical equilibrium models. Environ Sci Technol.

[CR40] Meyer R, Engesgaard P, Sonnenborg TO (2019). Origin and dynamics of saltwater intrusion in a regional aquifer: combining 3-D saltwater modeling with geophysical and geochemical data. Water Resour Res.

[CR41] Michael HA, Mulligan AE, Harvey CF (2005). Seasonal oscillations in water exchange between aquifers and the coastal ocean. Nature.

[CR42] Nielsen DR, Biggar JW (1962). Miscible displacement: 3. Theoretical considerations. Soil Sci Soc Am Proc.

[CR43] Nkedi-Kizza P, Rao PSC, Jessup RE, Davidson JM (1982). Ion exchange and diffusive mass transfer during miscible displacement through an aggregated Oxisol. Soil Sci Soc Am J.

[CR44] Nkedi-Kizza P, Biggar JW, van Genuchten MT, Wierenga PJ, Selim HM, Davidson JM, Nielsen DR (1983). Modeling tritium and chloride 36 transport through an aggregated Oxisol. Water Resour Res.

[CR45] Nkedi-Kizza P, Biggar JW, Selim HM, van Genuchten MT, Wierenga PJ, Davidson JM, Nielsen DR (1984). On the equivalence of two conceptual models for describing ion exchange during transport through an aggregated Oxisol. Water Resour Res.

[CR46] Ozaki S, Akl CA, Nagino T, Hiroshiro Y (2021). Investigating effect of pumping ratio on effectiveness of barrier wells for saltwater intrusion: lab-scale experiments and numerical modeling. Water.

[CR47] Paldor A, Michael HA (2021). Storm surges cause simultaneous salinization and freshening of coastal aquifers, exacerbated by climate change. Water Resources Research.

[CR48] Parker JC, van Genuchten MT (1984) Determining transport parameters from laboratory and field tracer experiments. Bulleton 3, Virginia Agricultural Experimental Station. Blacksburg, VA, 91

[CR49] Passioura JB (1971). Hydrodynamic dispersion in aggregated media: 1. Theory Soil Sci.

[CR50] Passioura JB, Rose DA (1971). Hydrodynamic dispersion in aggregated media: 2. Effects of velocity and aggregate size. Soil Sci.

[CR51] Peaceman DW, Rachford HH (1962). Numerical calculation of multidimensional miscible displacement. Soc Petrol Eng J.

[CR52] QNV (2030) Qatar National Vision 2030. 15 May 2022 at https://www.gco.gov.qa/en/about-qatar/national-vision2030/

[CR53] Rao PSC, Rolston DE, Jessup RE, Davidson JM (1980). Solute transport in aggregated porous media: theoretical and experimental evaluation. Soil Sci Soc Am J.

[CR54] Robertson RHS (1957) Sepiolite: a versatile raw material. Chemistry and Industry 1492–1495

[CR55] Rose DA, Passioura JB (1971). The analysis of experiments on hydrodynamic dispersion. Soil Sci.

[CR56] Rose DA, Garratt JA, van Beinum W, Adey MA (2008). Modelling the leaching of ions from a structured porous material. J Agric Sci - Cambridge.

[CR57] Rose DA, Abbas F, Adey MA (2009). The effect of surface–solute interactions on the transport of solutes through porous materials. Eur J Soil Sci.

[CR58] Selim HM, Liwang M (1995). Transport of reactive solutes in soils: a modified two-region approach. Soil Sci Soc Am J.

[CR59] Seyfried MS, Rao PSC (1987). Solute transport in undisturbed columns of an aggregated tropical soil: preferential flow effects. Soil Sci Soc Am J.

[CR60] Sherif MM, Hamza KI (2001). Mitigation of seawater intrusion by pumping brackish water. Transp Porous Media.

[CR61] Shi W, Lu C, Werner AD (2020). Assessment of the impact of sea-level rise on seawater intrusion in sloping confined coastal aquifers. J Hydrol.

[CR62] Slama F (2010) Field experimentation and modelling of salts transfer in Korba coastal plain: Impact of seawater intrusion and irrigation practices. Ph.D. thesis, Neuchatel University, Centre of Hydrogeology 112

[CR63] Stein S, Yechieli Y, Shalev E, Kasher R, Sivan O (2019). The effect of pumping saline groundwater for desalination on the fresh–saline water interface dynamics. Water Resour.

[CR64] Stein S, Sola F, Yechieli Y, Shalev E, Sivan O, Kasher R, Vallejos A (2020). The effects of long-term saline groundwater pumping for desalination on the fresh–saline water interface: Field observations and numerical modeling. Sci Total Environ.

[CR65] Stein S, Michael HA, Dugan B (2021). Injection of desalination brine into the saline part of the coastal aquifer; environmental and hydrological implications. Water Res.

[CR66] Tiwari P, Chandak R, Yadav RK (2014). Effect of salt water on compressive strength of concrete. Int J Eng Res Appl.

[CR67] Toride N, Leij FJ, van Genuchten MT (1993). A comprehensive set of analytical solutions for nonequilibrium solute transport with first-order decay and zero-order production. Water Resour Res.

[CR68] Toride N, Leij FJ, van Genuchten MT (1995) The CXTFIT Code for estimating transport parameters from laboratory or field tracer experiments (version 2.0). Research Report No. 137, US Salinity Laboratory, Riverside, CA

[CR69] van Beinum W (2007) Modelling multicomponent solute transport in structured soils. PhD thesis, Wageningen University, Wageningen, The Netherlands. 177

[CR70] van Genuchten MT, Wierenga PJ (1986) Solute dispersion coefficients and retardation factors. In A. Klute (ed.) Methods of soil analysis. Part 1 Physical and mineralogical methods. Second edition. SSSA, Madison, WI

[CR71] van Genuchten MT, Wierenga PJ (1976). Mass transfer studies in sorbing porous media: 1. Analytical solutions. Soil Sci Soc Am J.

[CR72] Vanderborght J, Vereecken H (2007). Review of dispersivities for transport modelling in soils. Vadose Zone Journal.

[CR73] Walther M, Stoekl L, Morgan LK (2020). Post-pumping seawater intrusion at the field scale: implications for coastal aquifer management. Adv Water Resour.

[CR74] Werner AD, Simmons CT (2009). Impact of sea-level rise on sea water intrusion in coastal aquifers. Groundwater.

[CR75] World Health Organization (WHO) (2004). Guidelines for drinking-water quality, recommendations.

[CR76] Wu WY, Lo MH, Wada Y, Famiglietti JS, Reager JT, Yeh PJF, Ducharne A, Yang ZL (2020). Divergent effects of climate change on future groundwater availability in key mid-latitude aquifers. Nat Commun.

[CR77] Yu X, Michael HA (2019). Mechanisms, configuration typology, and vulnerability of pumping-induced seawater intrusion in heterogeneous aquifers. Adv Water Resour.

[CR78] Zghibi A, Tarhouni J, Zouhri L (2013). Assessment of seawater intrusion and nitrate contamination on the groundwater quality in the Korba coastal plain of Cap-Bon (north east of Tunisia). J African Earth Sci.

[CR79] Zhou Q, Bear J, Bensabat J (2005). Saltwater upcoming and decay beneath a well pumping above an interface zone. Transport Porous Media.

